# Observations on the Modus Operandi of Calomel

**Published:** 1832

**Authors:** 


### Art. III. *Observations on the Modus Operandi of Calomel.* By a Correspondent.

The following letter to the junior Editor was not intended for publication, but the views it offers of the modus operandi of a medicine so much in use, appear to us so rational, that we take the liberty of offering them to the public. Correct philosophy has an indirect, indeed, but a powerful bearing upon judicious practice; and cannot, either in medicine or in other sciences, be too highly appreciated.

*Dear Sir*,—I ought to apologise for not sooner fulfilling my promise to write to you on the subject of *Calomel*. My attention has been so occupied by other matters, that I had nearly forgotten my promise—though to you it cannot make much difference, as I before stated to you, since I possess but a very limited knowledge of the subject. As you wished to know, however, in what light I regarded calomel, I offer the following as a summary of the views which I entertain upon the subject.

Calomel, as a *purgative*, commences its operation high up, acting forcibly on the stomach and duodenum.

It promotes and modifies the secretion of the liver and pancreas, as well as those of the mucous membrane of the intestinal tube throughout.

It appears in some instances, to be capable of abating irritation, and of overcoming mordid action in the alimentary canal, independently of the evacuation of vitiated matters, and in a manner analogous to that of direct sedatives; as, for example, in dysentery, in diarrhœa, and cholera: at least in certain cases of these diseases. (Ainsley, Johnson, Cartwright, &c.)

When given in large doses, besides its primary action on the bowels, it produces its specific effects on the general system, called the mercurial impression: and this, whatever may be its nature, seems more especially to affect the capillary vessels, and the receiving and absorbing systems. By exciting the action of these parts, it has a tendency to equalize the general circulation—restore secretions, overcome local congestion, inflammation, &c. This mercurial impression, carried to a greater extent, causes ptyalism, which, besides being the surest evidence that a mercurial action is set up in the system, is of itself, probably a salutary local disease.

The natural tendency of the mercurial action is to prostrate, and to derange, more or less, the constitution; but when it is opposed to a formidable disease, besides overcoming a greater evil than itself, it seems probable, that its own injurious tendency may be in a measure neutralized by the disease which it overcomes. Still, a decided mercurial action should not be resorted to on slight occasions, as if it were always a harmless expedient. I subscribe to Dr. Armstrong's opinion, that it is safest in diseases of febrile action, or of congestion, and when there is no great prostration; and that it is apt to do injury in cases of debility, of low action, and in constitutions naturally feeble or unsound; as its tendency in such cases is to prostrate the system, or to awaken some latent disease, and impair the general health.

Does mercury enter the circulation; or does its constitutional effect depend on sympathy alone? I believe it is absorbed into the circulation, but shall not trouble you with my reasons for this belief.

Again, how does mercury overcome disease? Is Boerhaave, Cullen, or John Hunter, right in his hypothesis? The last is very plausible, and so is Mr. Teale (of Leeds). He believes that mercury pervades the circulation, where it operates as a stimulant, not so much, however, on the central, as it does on the extreme vessels, which it excites into brisk action. If they are overloaded with blood from congestion or inflammation, their caliber is diminished, and their healthy condition restored. In short, he holds that it operates on the same principles as external stimuli do upon an inflamed part, except that the mercury makes its impression, ab interno. In this way he explains the beneficial effects of mercury in membranous inflammation, and in restoring a due circulation in parts where it is deficient.

It has also been discovered, that the blood undergoes a change, during the action of mercury—that it is rendered less consistent—and that the proportion of crassamentum is lessened, and that the albumen of the blood and of the secretions is changed, in some measure, to a serous character, (Bostock, Goss and Travers). These are important discoveries, and may throw much light on this obscure subject—and it justifies the belief, that we are, as yet, very imperfectly acquainted with the modus operandi of mercury in curing diseases.

In estimating the share of credit due to mercurial action, especially in fevers, we should be careful not to award to it the glory of a victory, when it has only been *permitted* to take possession of the field, because the enemy had chosen to retire. Nor should the novice in medicine be unapprized of the possibility of his placing to the account of the disease, derangements which are caused solely by the mercury, with which he is plying his patient. I might make further trite remarks on calomel and mercury, but the above exhibits a general outline of my views upon the subject.

